# Liver Injury Repair and Regeneration: Molecular Mechanisms and Therapeutic Targets

**DOI:** 10.1002/mco2.71108

**Published:** 2026-11-01

**Authors:** Mingyue Pan, Ni An, Yutian Feng, Yuelei Hu, Chongqin Pan, Yuxin Su, Yuanzheng Huang, Tanqing Long, Qi Wang, Qingru Song, Pengfei Li, Xiaomeng Li, Wenzhen Yin, Zhiyuan Hu, Xiaoxuan Liu, Juan Liu

**Affiliations:** ^1^ School of Future Medicine Beijing University of Chinese Medicine Beijing China; ^2^ State Key Laboratory of Natural Medicines and Jiangsu Key Laboratory of Drug Discovery for Metabolic Diseases Center of Advanced Pharmaceuticals and Biomaterials China Pharmaceutical University Nanjing China; ^3^ Clinical Translational Science Center Beijing Tsinghua Changgung Hospital Tsinghua University Beijing China; ^4^ Department of Biomedical Engineering School of Biomedical Engineering Tsinghua University Beijing China; ^5^ Department of Hepatobiliary and Pancreatic Surgery General Surgery Center First Hospital of Jilin University Changchun China; ^6^ School of Pharmacy Tongji Medical College Huazhong University of Science and Technology Wuhan China; ^7^ CAS Key Laboratory for Biomedical Effects of Nanomaterials and Nanosafety CAS Key Laboratory of Standardization and Measurement for Nanotechnology CAS Center for Excellence in Nanoscience National Center for Nanoscience and Technology Beijing China

**Keywords:** clinical translation, liver injury repair, liver regeneration, nanocarrier‐based delivery, regenerative microenvironment

## Abstract

The liver possesses a remarkable regenerative capacity to restore tissue integrity and physiological function following acute injury. However, this endogenous repair potential is gradually impaired in chronic liver diseases, including metabolic dysfunction–associated steatohepatitis (MASH) and hepatic fibrosis. These disorders frequently lead to persistent inflammation and progressive hepatic dysfunction. Our incomplete understanding of the mechanisms behind pathological liver regeneration limits the development of new regenerative therapies. This review summarizes recent advances in liver regeneration across temporal, cellular, metabolic, and interorgan communication aspects. It describes the dynamic features of different regenerative phases and emphasizes the regulatory roles of non‐parenchymal cells within the regenerative niche. We also outline promising translational strategies, including molecular‐targeted therapy, cell‐based therapy, liver tissue engineering, and nanocarrier delivery systems. This review links basic mechanistic findings to clinical translation, discusses key existing research challenges, and offers useful insights for developing precision therapeutics for liver regeneration.

## Graphical abstract

This review outlines the spatiotemporal framework of liver injury repair and regeneration, orchestrated by molecular signaling, multicellular niche crosstalk, metabolic reprogramming, and interorgan communication. Post‑injury regenerative cascades (initiation, proliferation, termination) rely on crosstalk between hepatocytes, Kupffer cells, hepatic stellate cells, liver sinusoidal endothelial cells, and immune populations, additionally modulated by liver–gut, –spleen, –kidney, –brain, and –adipose axes. We highlight emerging therapies including targeted small molecules, cell–tissue engineering, and stimuli‑responsive nanodelivery. Overcoming pathological heterogeneity and improving liver‑targeted strategies will facilitate translation toward precision liver regenerative medicine.

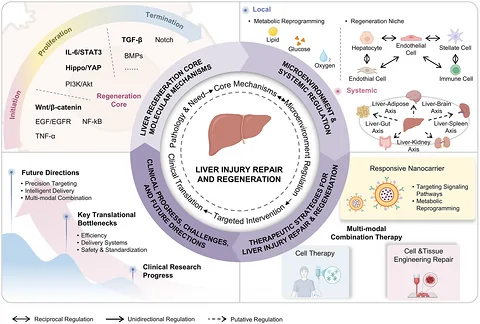

## 1 Introduction

The liver, as a central metabolic, detoxification, and biosynthetic organ, possesses remarkable regenerative capacity after injury [1]. However, liver regeneration is not a passive process but a highly coordinated spatiotemporal program regulated by interconnected signaling networks, cellular interactions, metabolic remodeling, regenerative microenvironment, and systemic factors. The molecular mechanisms governing successful regeneration versus regenerative failure remain incompletely understood [2, 3].

Liver diseases associated with injury, including viral hepatitis, alcohol‐associated liver disease, and metabolic dysfunction–associated steatohepatitis (MASH), represent a growing global health burden. Among these disorders, MASH has emerged as a major driver of chronic liver injury, largely attributed to the increasing prevalence of obesity and metabolic syndrome worldwide [4, 5]. Meanwhile, the growing prevalence of end‐stage liver disease, together with the shortage of donor organs, immune rejection, and long‐term complications associated with transplantation, highlights the urgent need for therapeutic strategies beyond organ replacement [6].

Current clinical approaches mainly focus on etiological intervention, hepatoprotection, and management of disease complications. Although these strategies can delay disease progression, they remain insufficient to restore damaged hepatic architecture and regenerative capacity in advanced fibrosis and cirrhosis [7]. In acute liver failure and extensive hepatectomy, the timely initiation and proper execution of regenerative responses directly determine patient outcomes. In chronic liver diseases, impaired regenerative capacity and dysregulated regenerative timing contribute to persistent inflammation, tissue remodeling, and progressive hepatic dysfunction [8, 9]. Therefore, precisely understanding and therapeutically manipulating endogenous liver regeneration represents a critical unmet clinical need.

In this review, we provide an integrated overview of liver injury repair and regeneration from temporal, cellular, metabolic, and systemic perspectives (Figure 1). As illustrated in the figure, this framework connects mechanistic insights with translational strategies across multiple scales. We summarize the sequential regenerative phases, including injury sensing, initiation, proliferation, and termination, and discuss how signaling networks, non‐parenchymal cells, metabolic reprogramming, regenerative microenvironment remodeling, and multi‐organ communication collectively regulate regenerative outcomes. Furthermore, we highlight emerging therapeutic strategies, including pathway‐targeted interventions, cell‐based therapies, tissue engineering, responsive nanodelivery systems, and multimodal approaches, with emphasis on translational advances, current challenges, and future opportunities.

**FIGURE 1:**
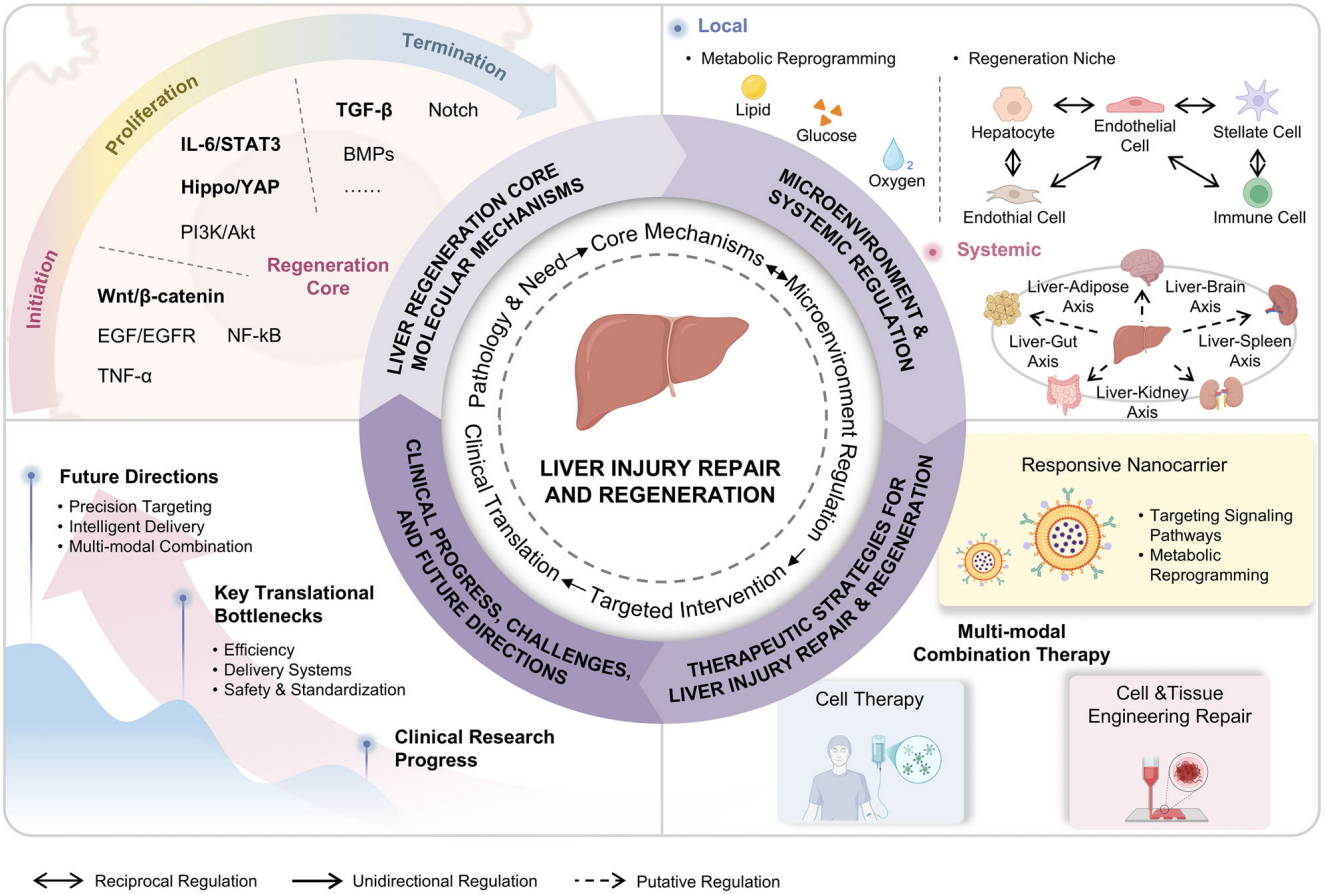
Integrated framework of liver injury repair and regeneration: from molecular mechanisms to clinical translation. Overview of the conceptual framework of liver injury repair and regeneration, integrating four interconnected aspects: molecular mechanisms underlying regenerative regulation, local and systemic microenvironmental interactions, emerging therapeutic strategies, and clinical translation challenges and opportunities. The framework highlights the transition from mechanistic understanding to targeted interventions and precision regenerative therapies. Solid arrows, bidirectional arrows, and dashed arrows indicate directional regulation, reciprocal interactions, and putative regulatory relationships, respectively.

## 2 Temporal Regulation of Liver Regeneration in Three Phases

Following liver injury, the liver activates a highly coordinated regenerative program to restore tissue mass and maintain functional homeostasis [10]. Liver regeneration is a dynamic spatiotemporal process regulated by sequential activation, amplification, and termination of regenerative signals involving inflammatory responses, growth factor signaling, metabolic adaptation, and microenvironmental remodeling [11, 12].

Despite variations among different injury models, the core regenerative process can be broadly divided into three sequential phases: initiation, proliferation, and termination. Each phase is governed by distinct molecular programs and coordinated transitions to ensure appropriate restoration of liver structure and function [11, 12]. This section summarizes the temporal regulation of liver regeneration and highlights the key signaling networks involved in phase‐specific progression.

### 2.1 Initiation Phase: Injury Sensing and Regenerative Priming

The initiation phase (0–6 h after injury) represents the transition from tissue damage recognition to regenerative activation. During this stage, hepatocytes and non‐parenchymal cells rapidly sense injury‐associated signals and establish an inflammatory microenvironment required for regenerative priming. Damage‐associated molecular patterns (DAMPs) and pathogen‐associated molecular patterns (PAMPs) activate Kupffer cells, inducing NF‐κB signaling and promoting secretion of pro‐inflammatory cytokines, particularly TNF‐α and IL‐6 [13, 14].

TNF‐α induces negative regulators including A20 and SOCS1/3 to prevent excessive inflammatory activation, whereas IL‐6 activates the JAK/STAT3 pathway and promotes hepatocytes entering a proliferative‐competent state [15]. In addition to inflammatory signals, non‐parenchymal cells contribute to regenerative initiation through paracrine regulation. Hepatic stellate cells (HSCs), for example, release pro‐HGF, VEGF, and other factors, thereby coordinating multiple signaling pathways involved in early regeneration [15, 16].

The regulatory mechanisms during initiation vary depending on injury context. Molecules such as IL‐17 and Nrf2 further contribute to maintaining the transition from injury sensing toward the proliferative phase, ensuring continuous progression of the regenerative response [17, 18].

### 2.2 Proliferative Phase: Signal‐Driven Hepatocyte Expansion

The proliferative phase (6–72 h after injury) is characterized by extensive hepatocyte expansion and is primarily regulated by coordinated activation of IL‐6/STAT3, HGF/c‐MET, EGF/EGFR, and Hippo‐YAP/TAZ signaling pathways [19, 20].

STAT3 promotes transcription of regeneration‐associated genes and suppresses hepatocyte apoptosis, whereas HGF and EGF stimulate hepatocyte growth mainly through activation of the PI3K/Akt pathway [21, 22, 23]. Meanwhile, suppression of Hippo pathway activity facilitates YAP/TAZ nuclear translocation, inducing downstream genes associated with proliferation and cell survival [24, 25]. These pathways form an interconnected signaling network that amplifies regenerative responses.

Efficient hepatocyte proliferation requires coordinated metabolic adaptation and microenvironmental support. The PI3K/Akt/mTOR pathway regulates metabolic reprogramming to provide sufficient resources for cell division [26]. Meanwhile, polarization of monocyte‐derived macrophages toward anti‐inflammatory phenotypes helps reduce inflammatory interference with cell‐cycle progression [27]. β1‐Integrin integrates extracellular matrix signals with growth factor signaling to maintain proliferative stability [28]. In parallel, PTEN negatively regulates PI3K/Akt signaling to prevent excessive activation, whereas the Ras/Raf/MEK/ERK pathway promotes proliferation under intrinsic feedback control [29, 30].

### 2.3 Termination Phase: Cessation of Proliferation and Restoration of Tissue Homeostasis

Following restoration of liver mass to near‐physiological levels (approximately 72 h after injury), regeneration transitions into the termination phase, during which proliferative signals are attenuated and tissue homeostasis is reestablished [31].

TGF‐β and Hippo signaling are central regulators of regenerative termination. TGF‐β derived from HSCs and Kupffer cells induces cell‐cycle inhibition through downstream Smad2/3 signaling, whereas increased cell density activates Hippo signaling and suppresses YAP/TAZ activity. These mechanisms collectively restrict further hepatocyte proliferation and terminate the regenerative program [32].

BMP and Notch signaling contribute to proliferation restraint and biliary epithelial remodeling, while caspase‐3‐mediated physiological apoptosis eliminates excessive hepatocytes to maintain appropriate cell numbers [33, 34]. Notably, in chronic liver injury, persistent TGF‐β activation promotes fibrosis, and disruption of regenerative signaling balance contributes to impaired endogenous repair capacity [35].

### 2.4 Key Regulatory Nodes Governing Phase Transition

The orderly progression of liver regeneration depends on precise coordination among signaling thresholds, metabolic states, and microenvironmental changes. The transition from initiation to proliferation requires appropriate activation and subsequent attenuation of inflammatory signaling, including TNF‐α/NF‐κB and IL‐6/STAT3 pathways, together with the engagement of growth factor‐mediated regenerative signals such as HGF and EGF [36, 37].

The transition from proliferation to termination is primarily driven by restoration of liver mass, increased hepatocyte density, accumulation of inhibitory signals such as TGF‐β, and metabolic adaptation from proliferative programs toward tissue homeostasis [38]. Dysregulation of these regulatory nodes, including persistent YAP activation or impaired TGF‐β signaling balance, may disrupt regenerative termination, promote fibrosis progression, and increase the risk of malignant transformation [32].

Collectively, liver regeneration is governed by a dynamic spatiotemporal regulatory network, in which inflammatory activation, growth factor signaling, metabolic adaptation, and microenvironmental remodeling are sequentially coordinated to ensure efficient tissue repair while preventing excessive proliferation. The major molecular events and regulatory interactions across the initiation, proliferation, and termination phases are summarized in Figure 2.

**FIGURE 2:**
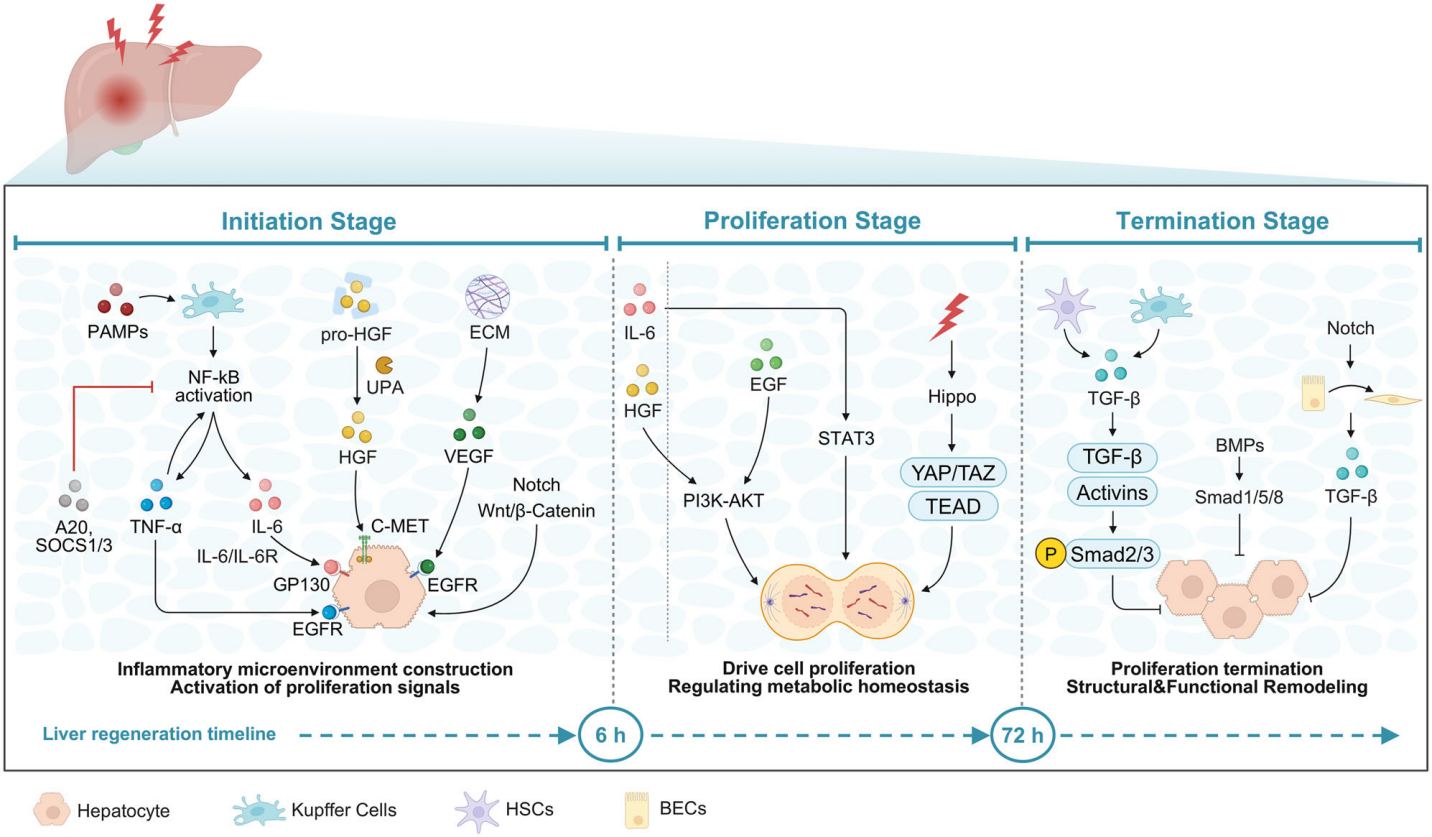
Spatiotemporal molecular regulatory network of liver regeneration. Schematic overview of the molecular and cellular events during the initiation, proliferation, and termination phases of liver regeneration. The figure illustrates the sequential regulation of injury sensing, inflammatory activation, regenerative signaling, hepatocyte proliferation, and restoration of tissue homeostasis through coordinated interactions among hepatocytes, Kupffer cells, hepatic stellate cells, and biliary epithelial cells. BECs, biliary epithelial cells; BMPs, bone morphogenetic proteins; EGF, epidermal growth factor; HGF, hepatocyte growth factor; HSCs, hepatic stellate cells; TGF‐β, transforming growth factor β.

## 3 Core Signaling Regulatory Networks in Liver Regeneration

The regenerative response after liver injury is governed by an interconnected signaling network that integrates inflammatory activation, growth factor stimulation, metabolic adaptation, and mechanical cues. These pathways coordinate hepatocyte proliferation, survival, and tissue remodeling in a highly spatiotemporal manner rather than functioning as isolated regulatory modules [39]. This section summarizes the major signaling pathways involved in liver regeneration and their coordinated interactions across different regenerative phases.

### 3.1 Major Pro‐Regenerative Signaling Pathways

#### 3.1.1 VEGF/VEGFR Signaling Pathway

Angiogenesis is an essential component of liver regeneration, and VEGF/VEGFR signaling contributes to vascular remodeling from the initiation to proliferative phases [40]. Following liver injury, VEGF‐A expression is increased in hepatocytes, Kupffer cells, and HSCs. VEGF‐A binding to VEGFR‐2 on liver sinusoidal endothelial cells activates PI3K/Akt and ERK/MAPK signaling, thereby promoting endothelial proliferation, migration, and sinusoidal reconstruction to support parenchymal regeneration [41].

Beyond vascular remodeling, endothelial cells function as an important regenerative niche by releasing HGF and Wnt2 through paracrine mechanisms, thereby promoting hepatocyte proliferation and biliary epithelial cell reprogramming [40]. VEGF/VEGFR activity is tightly regulated in a temporal manner, with early activation followed by attenuation during regeneration termination, ensuring coordinated vascular and parenchymal restoration [42].

#### 3.1.2 Wnt/β‐Catenin Signaling Pathway

The Wnt/β‐catenin pathway is a central driver of hepatocyte proliferation, particularly during the proliferative phase. Wnt ligands, including Wnt2 and Wnt9b derived from liver sinusoidal endothelial cells, activate β‐catenin signaling, leading to nuclear translocation and induction of proliferation‐associated genes such as c‐Myc and cyclin D1 [43].

The regenerative function of Wnt/β‐catenin signaling depends on injury context. Mild injury mainly promotes mature hepatocyte replication, whereas severe injury enhances hepatic progenitor cell activation. Moreover, interactions with PI3K/Akt, PPARγ, and Notch pathways contribute to maintaining regenerative balance and phase‐specific responses [44, 45].

#### 3.1.3 PI3K/Akt/mTOR Signaling Pathway

The PI3K/Akt/mTOR pathway acts as a central signaling hub linking extracellular growth signals with intracellular proliferation and metabolic adaptation. Activation by factors such as HGF and EGF promotes Akt phosphorylation, which suppresses apoptosis, facilitates cell‐cycle progression, and activates mTORC1‐dependent biosynthetic programs required for hepatocyte expansion [20].

Importantly, this pathway integrates proliferative demand with cellular energy status. Under metabolic stress, AMPK‐mediated inhibition of mTORC1 prevents excessive proliferation, highlighting its role as a metabolic checkpoint during regeneration [37, 46].

#### 3.1.4 Hippo/Yap Signaling Pathway

The Hippo/YAP pathway serves as a critical mechanotransduction system that senses tissue tension and cell density during liver regeneration [47]. Early after injury, reduced mechanical stress suppresses MST1/2‐LATS1/2 activity, allowing nuclear accumulation of YAP/TAZ and activation of proliferation‐related genes. YAP/TAZ cooperates with Wnt and PI3K/Akt signaling to enhance regenerative responses [25].

After restoration of liver mass, cell–cell contact activates Hippo signaling, promoting YAP/TAZ phosphorylation and degradation to terminate proliferation. In addition, interaction between Hippo/YAP and TGF‐β/Smad signaling contributes to the transition from proliferation toward tissue homeostasis [31, 37].

#### 3.1.5 NF‐κB Signaling Pathway

NF‐κB represents a key inflammatory signaling pathway during the initiation phase of liver regeneration. Following injury, IKK activation induces IκB degradation, enabling NF‐κB nuclear translocation and transcriptional regulation of inflammatory and survival‐related genes [36, 48]. This process establishes an inflammatory environment required for regenerative activation [49].

NF‐κB functions in a cell‐type‐dependent manner: Hepatocyte NF‐κB primarily promotes survival and antiapoptotic responses, whereas Kupffer cell‐derived NF‐κB signaling enhances inflammatory mediator production. This balance allows appropriate inflammatory activation without excessive tissue damage [50, 51].

#### 3.1.6 STAT3 Signaling Pathway

STAT3 serves as a critical bridge between inflammatory signaling and hepatocyte proliferation during the initiation and proliferative phases. IL‐6 and EGF stimulation activates JAK‐dependent STAT3 phosphorylation, leading to nuclear translocation and transcriptional regulation of regeneration‐associated genes [21, 52].

During proliferation, sustained STAT3 activation supports hepatocyte expansion and metabolic adaptation, while forming regulatory interactions with NF‐κB and PI3K/Akt/mTOR signaling to amplify regenerative responses [53].

#### 3.1.7 Crosstalk and Synergistic Networks Among Signaling Pathways

Liver regeneration is controlled by coordinated pathway networks rather than independent signaling cascades. During initiation, NF‐κB and STAT3 establish an inflammatory activation module; during proliferation, Wnt/β‐catenin and growth factor pathways drive hepatocyte expansion; whereas Hippo/YAP and TGF‐β/Smad signaling contribute to proliferation termination and tissue restoration [36].

Shared regulatory nodes, including GSK‐3β and β‐catenin, integrate signals from multiple pathways and enhance network robustness [20, 53]. Meanwhile, these signaling interactions are further shaped by the regenerative microenvironment established by non‐parenchymal cells, which will be discussed in subsequent sections. The major pathways and their regulatory features are summarized in Table 1.

**TABLE 1:** Summary of core proliferative and survival signaling pathways involved in liver regeneration.

Signaling pathway	Core effector molecules	Primary biological functions	Active regenerative stages	Cross‐regulation with other pathways	Reference
VEGF/VEGFR pathway	VEGFR2, Akt, ERK	Promote angiogenesis and vascular remodeling to support liver regeneration	Initiation stage, proliferation stage	Crosstalk with PI3K/Akt	[19, 54, 55]
Wnt/β‐catenin pathway	β‐catenin, TCF/LEF	Drive hepatocyte cell cycle progression; modulate cell differentiation fate	Proliferation stage	Synergize with PI3K/Akt; antagonize Notch pathway	[20, 44, 56]
PI3K/Akt/mTOR pathway	Akt, mTORC1	Inhibit apoptosis, facilitate cell cycle progression, and regulate synthetic metabolism	Initiation stage, proliferation stage	Negatively regulated by AMPK; form synergistic axis with STAT3	[20, 54, 57]
Hippo/YAP pathway	YAP/TAZ, LATS1/2	Sense tissue mechanical cues, control organ size, and terminate proliferative programs	Initiation stage, proliferation stage	Synergize with Wnt and PI3K/Akt; couple with TGF‐β signaling	[20, 58, 59]
NF‐κB pathway	p65/p50, IKK	Rapidly respond to hepatic injury, initiate early inflammatory response, and exert hepatoprotective effects	Initiation stage	Activate STAT3 to constitute the early inflammatory initiation module	[37, 56, 60]
STAT3 pathway	STAT3, JAK	Bridge inflammatory signals and proliferative programs; sustain hepatocyte survival and proliferation	Initiation stage, proliferation stage	Form synergistic signaling network with NF‐κB and PI3K/Akt	[37, 61, 62]

### 3.2 Signaling Dysregulation and Imbalance in Chronic Liver Injury

Chronic liver injury, including MASH, disrupts the spatiotemporal regulation of regenerative signaling networks. Persistent pathological stimuli impair physiological repair programs, ultimately resulting in defective endogenous regeneration [19].

Chronic inflammation represents a major driver of regenerative failure. Sustained inflammatory activation alters the balance of key signaling pathways, among which excessive TGF‐β signaling promotes fibrosis progression and disrupts regenerative microenvironment homeostasis through persistent activation of HSCs and fibrogenic responses [63].

Metabolic dysregulation further compromises regenerative capacity in metabolic dysfunction–associated steatotic liver disease. Hepatic lipid accumulation interferes with energy‐sensing pathways, leading to impaired proliferative signaling and reduced hepatocyte regenerative potential [63].

In addition, impaired interorgan communication, including dysregulation of the liver–gut and liver–spleen axes, contributes to regenerative defects. Intestinal endotoxin translocation, splenic immune alterations, and adipose‐derived lipotoxic factors collectively aggravate hepatic repair impairment through distinct mechanisms [64].

Together, these pathological alterations disrupt the balance between regenerative activation and termination control, resulting in insufficient proliferative responses and impaired tissue restoration. Such signaling imbalance represents a critical mechanism underlying the limited reversibility of advanced liver diseases through endogenous regeneration.

## 4 Regenerative Microenvironment and Cellular Interaction Regulation

The successful execution of liver regeneration requires not only coordinated signaling activation but also precise regulation by the regenerative microenvironment. This specialized niche, composed of non‐parenchymal cells, extracellular matrix components, and dynamic intercellular interactions, provides essential regulatory cues for hepatocyte activation, expansion, and restoration of tissue homeostasis [65, 66].

In this section, we summarize the major cellular components and interaction networks within the regenerative niche, highlighting how multicellular coordination shapes the initiation, proliferation, and termination phases of liver regeneration. The dynamic regenerative niche and its regulatory framework are illustrated in Figure 3.

**FIGURE 3:**
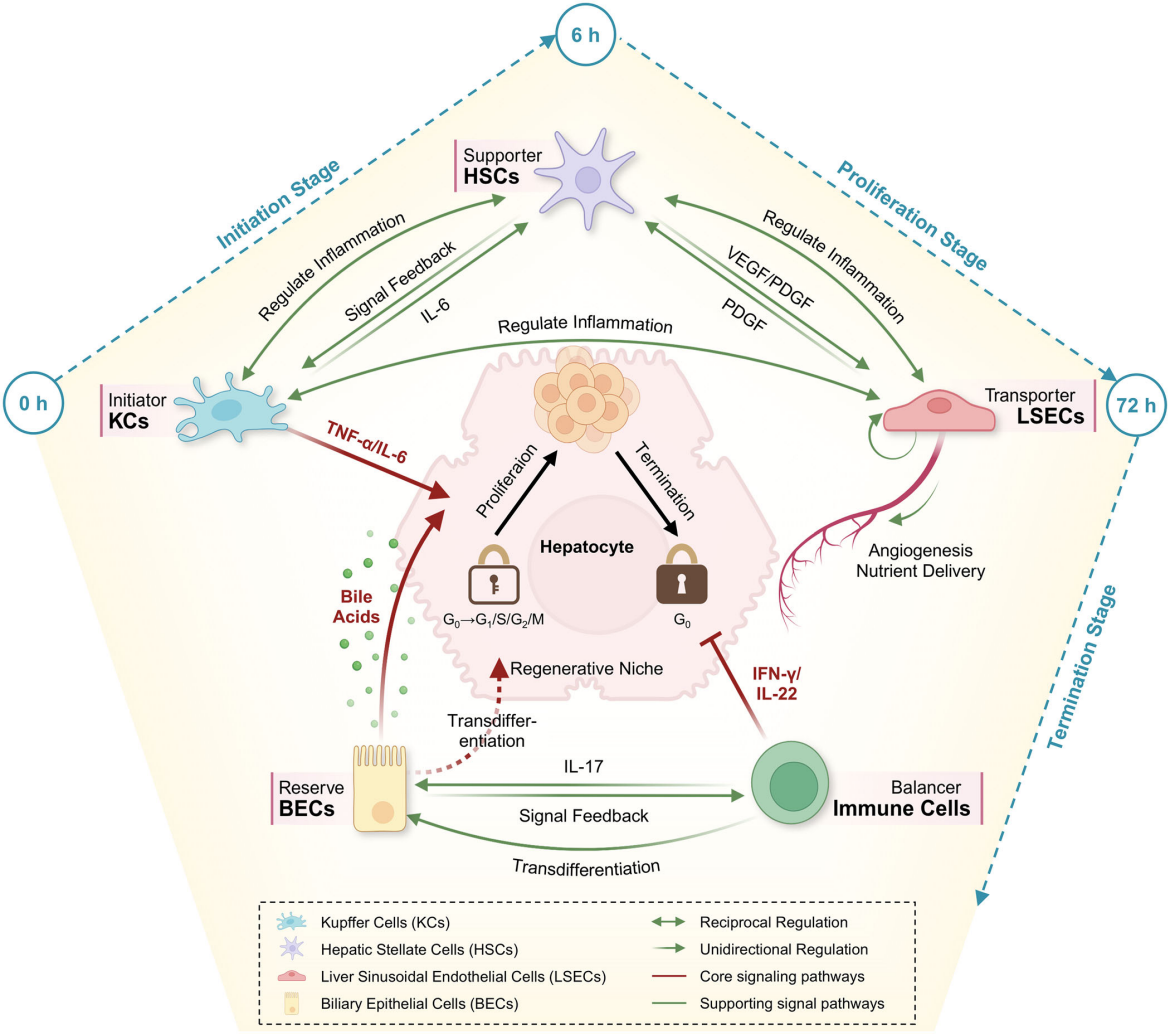
Dynamic regenerative niche regulated by multicellular coordination during liver regeneration. Kupffer cells (KCs), hepatic stellate cells (HSCs), liver sinusoidal endothelial cells (LSECs), biliary epithelial cells (BECs), and immune cell subsets collectively establish a dynamic regenerative niche through coordinated cellular interactions. During liver regeneration, these cellular populations regulate hepatocyte activation, proliferation, vascular remodeling, immune modulation, and restoration of tissue homeostasis in a phase‐dependent manner. The figure highlights the multicellular communication network underlying the initiation, proliferation, and termination phases of liver regeneration.

### 4.1 Functions and Division of Labor of Non‐Parenchymal Cells

Liver regeneration is not accomplished by hepatocytes alone but requires coordinated contributions from multiple non‐parenchymal cell populations. Kupffer cells, HSCs, and liver sinusoidal endothelial cells constitute major components of the regenerative niche. Through paracrine signaling and extracellular matrix remodeling, these cells establish an organized interaction network that collectively determines hepatocyte proliferative capacity, inflammatory responses, and tissue remodeling outcomes [67].

During different stages of liver regeneration, non‐parenchymal cells exhibit distinct temporal changes in functional states. During the initiation phase, they primarily mediate injury recognition and inflammatory activation. During the proliferative phase, they provide nutritional and structural support for hepatocyte expansion. During the termination phase, they contribute to the cessation of proliferation and restoration of tissue homeostasis. The coordinated functions and dynamic transitions of different non‐parenchymal cell populations are essential for maintaining the stability of the regenerative niche [68].

#### 4.1.1 Functions and Regulation of Major Non‐Parenchymal Cell Populations

##### 4.1.1.1 Kupffer Cells

Kupffer cells are key initiators of regenerative signaling after liver injury. Through activation of the TLR/MyD88 pathway, they release TNF‐α, IL‐6, and Wnt ligands, thereby activating NF‐κB/STAT3 and Wnt/β‐catenin signaling in hepatocytes to promote regenerative responses [14].

Kupffer cells exhibit marked phenotypic plasticity during regeneration. Early after injury, they adopt a pro‐inflammatory phenotype to initiate repair, whereas subsequent transition toward an anti‐inflammatory phenotype limits excessive inflammation and contributes to termination of regeneration through IL‐10 and TGF‐β signaling [69].

##### 4.1.1.2 HSCs

HSCs function as both structural regulators and signaling mediators within the regenerative niche [70]. Following activation after injury, they provide extracellular matrix support while secreting growth factors, including HGF and EGF, to stimulate hepatocyte proliferation through MAPK and PI3K‐Akt signaling [71, 72].

During later stages, HSCs contribute to termination of regeneration through secretion of inhibitory factors, including TGF‐β1 and BMPs, thereby coordinating the transition from proliferation toward tissue homeostasis [70, 73].

##### 4.1.1.3 Liver Sinusoidal Endothelial Cells

Liver sinusoidal endothelial cells serve as critical vascular regulators within the regenerative niche. Following injury, they sense vascular changes and release regenerative mediators, including Wnt2/9b and VEGF, to coordinate hepatocyte proliferation and angiogenic remodeling [74, 75, 76, 77].

Endothelial‐derived signals also promote recruitment of progenitor populations and enhance hepatocyte responsiveness to growth factors, establishing a positive feedback loop between vascular reconstruction and liver regeneration.

##### 4.1.1.4 Biliary Epithelial Cells

Biliary epithelial cells participate in both biliary repair and hepatocyte regeneration. Under conditions of impaired hepatocyte proliferation, they can contribute to restoration of the hepatocyte pool through transdifferentiation [78].

Biliary epithelial cell–derived signals, including bile acid–mediated FXR/TGR5 activation, regulate proliferative responses and cytokine balance [79]. In addition, YAP signaling and vascular regulatory factors coordinate biliary remodeling with hepatocyte replenishment and vascular support [80].

##### 4.1.1.5 Immune Cell Subsets

Immune cell populations maintain regenerative homeostasis by regulating inflammatory balance within the regenerative niche [74]. Monocyte‐derived macrophages undergo phenotypic transition from Ly6C^+^ to Ly6C^−^ states, accompanied by acquisition of anti‐inflammatory functions and induction of Wnt3a‐mediated biliary epithelial cell differentiation [81].

Other immune populations, including Th17/Treg cells, γδ T cells, NK cells, eosinophils, and innate lymphoid cells, regulate regeneration through cytokine networks involving IL‐17, IL‐22, and IL‐4 signaling [82, 83, 84]. Collectively, these immune components establish a finely tuned regulatory network that balances inflammation and repair.

#### 4.1.2 Intercellular Interaction Networks and Signaling Crosstalk

Non‐parenchymal cells communicate with hepatocytes and each other through coordinated paracrine networks, thereby integrating regenerative signals within the niche. Disruption of these interactions contributes to pathological regenerative failure.

During physiological regeneration, Kupffer cell‐derived IL‐6 stimulates HSCs to release HGF, promoting hepatocyte proliferation. Liver sinusoidal endothelial cells regulate hepatocyte activation through Wnt2/9b signaling while coordinating vascular remodeling via reciprocal PDGF/VEGF interactions with HSCs. In addition, biliary epithelial cells and Kupffer cells establish regenerative communication through OPN‐IL‐6 signaling [43, 85, 86].

Multiple signaling pathways cooperate within this cellular network. Wnt/β‐catenin, Hippo/YAP, Notch, and PI3K/Akt/mTORC1 pathways collectively regulate hepatocyte proliferation, progenitor differentiation, biliary remodeling, and metabolic adaptation [44, 87, 88, 89, 90]. Under chronic liver injury and MASH, persistent cellular activation disrupts this balance, leading to inflammatory imbalance, metabolic disturbance, and impaired regeneration.

### 4.2 Metabolic Reprogramming During Liver Regeneration

Metabolic reprogramming during liver regeneration is tightly integrated with inflammatory signals, mechanical cues, and nutritional status within the regenerative microenvironment. Through reciprocal regulation between signaling pathways and metabolic processes, hepatocytes and non‐parenchymal cells obtain sufficient energy and biosynthetic resources while maintaining regenerative timing [26].

#### 4.2.1 Microenvironment Signal‐Driven Regulation of Metabolic Reprogramming

Mechanical, inflammatory, and nutritional signals collectively shape metabolic transitions during liver regeneration.

Following injury, liver sinusoidal endothelial cells sense shear stress and activate AMPK signaling, promoting glycolysis and fatty acid oxidation, while the Notch1–KLF2 axis contributes to angiogenic metabolic regulation [91]. Meanwhile, TNF‐α and IL‐6 released by Kupffer cells regulate glucose metabolism through NF‐κB and STAT3 signaling, facilitating metabolic adaptation during regeneration [92].

Metabolic specialization across hepatic lobules further supports regeneration. Periportal hepatocytes preferentially utilize glycolysis and fatty acid synthesis to provide biosynthetic substrates, whereas pericentral hepatocytes rely more on fatty acid oxidation for energy maintenance. HIF‐1α and Wnt/β‐catenin signaling contribute to this zonated metabolic regulation [43].

#### 4.2.2 Metabolite‐Mediated Microenvironmental Signaling and Temporal Regulation

Metabolic intermediates function as signaling regulators that coordinate regenerative progression. Pyruvate promotes Kupffer cell‐derived pro‐regenerative signals through HIF‐1α stabilization, whereas arachidonic acid metabolites activate PI3K/Akt and ERK pathways to enhance repair responses. α‐Ketoglutarate regulates mTORC1 activity, and short‐chain fatty acids provide metabolic substrates required for cell division [2, 93].

Metabolic programs dynamically shift across regenerative phases. Early regeneration relies on glycolysis and fatty acid synthesis to support biosynthetic demands, whereas proliferative stages require enhanced glutamine metabolism to maintain mitochondrial function. During termination, restoration of fatty acid oxidation and PPARα signaling contributes to metabolic homeostasis and suppression of proliferative programs [2, 94].

In pathological states, this metabolic–signaling coupling becomes disrupted. Lipid accumulation in MASH suppresses AMPK activity and impairs regenerative signaling, whereas chronic inflammation and alcohol‐associated liver disease induce mitochondrial dysfunction and inhibit HGF/c‐MET signaling, ultimately limiting regenerative capacity [95].

## 5 Multi‐Organ Regulation of Liver Regeneration

Liver regeneration is not solely determined by intrahepatic mechanisms but is coordinated by systemic communication networks involving multiple organs, including the intestine, spleen, kidney, brain, and adipose tissue. These interorgan axes regulate regenerative outcomes through metabolic exchange, immune modulation, and neuroendocrine signaling, thereby influencing hepatocyte proliferation, inflammatory balance, and tissue remodeling [96].

This section summarizes the regulatory mechanisms of major interorgan axes and their potential implications for regenerative therapies. The integrated communication network among these organs is illustrated in Figure 4.

**FIGURE 4:**
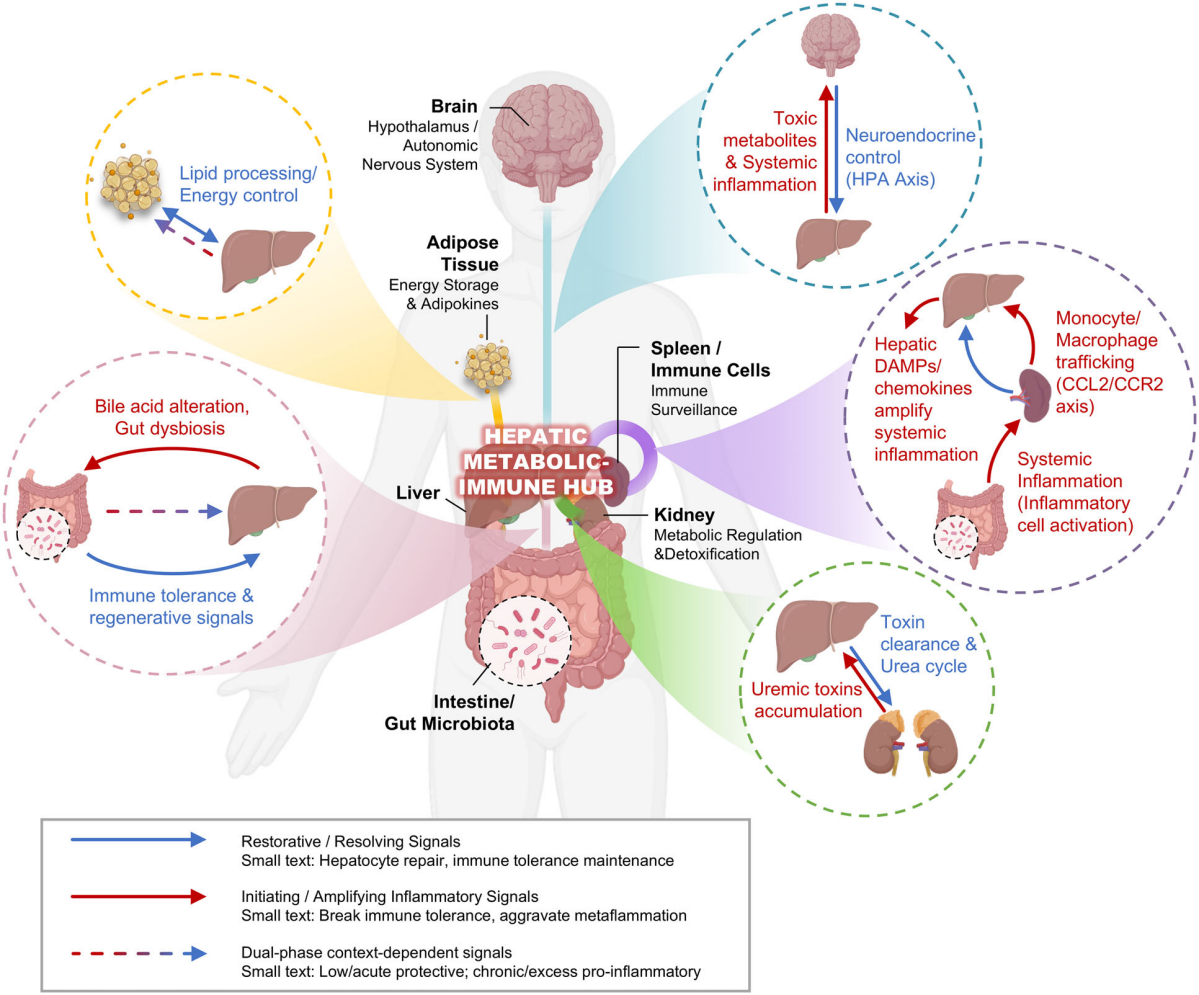
Multi‐organ communication networks shaping the hepatic regenerative microenvironment. The liver communicates with multiple organs, including the intestine, spleen, kidney, brain, and adipose tissue, through metabolic, immune, and neuroendocrine signals. Gut‐derived microbial metabolites regulate hepatic immune tolerance and regenerative signaling, whereas splenic immune cells modulate inflammatory balance during repair. The kidney contributes to metabolic homeostasis and detoxification, while brain‐derived neuroendocrine signals regulate hepatic metabolism and inflammation. Adipose tissue provides adipokines and metabolic substrates that influence regenerative capacity. Under pathological conditions, dysregulated interorgan communication promotes chronic inflammation and metabolic disturbance, impairing liver regeneration.

### 5.1 Liver–Gut Axis

The liver–gut axis regulates regeneration through microbial metabolites, immune signaling, and metabolic communication. Short‐chain fatty acids, secondary bile acids, and indole derivatives modulate regenerative responses by regulating inflammation and intracellular signaling pathways. During early regeneration, low‐grade lipopolysaccharide activates Kupffer cells to initiate repair responses, whereas short‐chain fatty acids promote hepatocyte proliferation through PI3K/Akt/mTOR signaling while maintaining intestinal barrier integrity [97, 98].

Gut microbiota dysbiosis disrupts this balance by increasing endotoxin translocation and metabolic stress, particularly in alcohol‐associated liver disease and metabolic‐associated liver diseases. Because hepatic bile acid metabolism and microbial metabolites reciprocally regulate each other, restoration of microbial homeostasis represents a potential strategy to improve the regenerative microenvironment [98, 99].

### 5.2 Liver–Spleen Axis

The liver–spleen axis is primarily mediated through portal circulation, enabling the transport of immune cells and soluble mediators between the two organs [100]. Following partial hepatectomy, splenic Ly6C^+^ monocytes migrate to the liver and promote hepatocyte proliferation through regenerative factors such as HGF and IL‐6, while splenic regulatory T cells contribute to immune homeostasis through anti‐inflammatory cytokine secretion [101].

The role of the spleen in liver regeneration is highly context‐dependent and exhibits bidirectional effects according to injury conditions. During partial hepatectomy with limited hepatocellular damage, splenic immune activation facilitates regenerative repair. However, in chronic liver diseases such as hepatitis and cirrhosis, splenic responses may instead promote inflammation and impair regeneration.

In cirrhosis with portal hypertension, splenic red pulp macrophages release TGF‐β1, which reaches the liver through portal circulation and continuously activates HSCs, thereby accelerating collagen deposition and fibrosis progression. Splenectomy may partially disrupt this profibrotic feedback loop and restore altered microbiota and metabolic homeostasis in cirrhotic patients [102, 103].

Mechanistically, portal hypertension–induced endotoxin accumulation promotes splenic macrophage polarization toward a pro‐inflammatory phenotype through TLR4 signaling. Although altered bile acid metabolism activates FXR‐mediated anti‐inflammatory responses, persistent endotoxin stimulation counteracts these protective effects. Furthermore, cirrhosis‐associated splenic immune dysfunction, including CD4^+^ T‐cell exhaustion, Th17/Treg imbalance, and impaired NK‐cell activity, reduces the clearance of activated HSCs and further suppresses regenerative capacity [104, 105].

### 5.3 Liver–Kidney Axis

Beyond immune regulation, the liver–kidney axis coordinates hepatic repair through metabolic, inflammatory, and neurohumoral interactions. Under physiological conditions, the kidney contributes to systemic metabolic homeostasis through gluconeogenesis, lactate clearance, and production of erythropoietin (EPO) and active vitamin D, thereby supporting liver regeneration [106, 107].

Disruption of this axis during kidney injury negatively affects hepatic repair. Acute kidney injury induces systemic inflammatory responses, whereas accumulation of uremic toxins and metabolic abnormalities during chronic kidney dysfunction aggravate hepatic inflammation and lipid disturbance, forming a vicious cycle between hepatic and renal impairment [108]. Accordingly, pharmacological modulation of liver–kidney communication, including sodium‐glucose cotransporter 2 (SGLT2) inhibitors and glucagon‐like peptide‐1 (GLP‐1) receptor agonists, may represent potential strategies to improve regenerative outcomes [109].

### 5.4 Liver–Brain Axis

At the neuroendocrine level, the liver–brain axis regulates hepatic regeneration through bidirectional neural and hormonal communication. Sympathetic and vagal pathways modulate hepatic metabolism and inflammation, while the hypothalamic–pituitary–adrenal axis contributes to immune regulation. Meanwhile, liver‐derived bioactive molecules influence central nervous system functions [110, 111, 112, 113].

In chronic liver diseases, excessive sympathetic activation and glucocorticoid elevation disturb hepatic microcirculation and suppress hepatocyte proliferation [113]. Impaired hepatic detoxification further promotes accumulation of neurotoxic metabolites, while gut microbiota dysbiosis contributes to dysfunction of the liver–gut–brain network [114]. Therefore, modulation of vagal activity and restoration of microbial homeostasis may provide potential approaches to simultaneously regulate neuroendocrine imbalance and hepatic inflammation [115, 116].

### 5.5 Liver–Adipose Axis

The liver–adipose axis regulates regeneration through adipokine signaling and metabolic substrate exchange. Under physiological conditions, adipose‐derived adiponectin and fibroblast growth factor 21 (FGF21) maintain hepatic metabolic homeostasis, whereas mobilized fatty acids provide energy and biosynthetic substrates for hepatocyte proliferation [117].

In contrast, obesity‐associated adipose inflammation disrupts this communication by increasing pro‐inflammatory mediator release, leading to hepatic insulin resistance and lipotoxic injury. Dysregulation of the liver–adipose axis further interacts with abnormalities in the liver–gut axis, creating a pathological feedback loop that impairs hepatic repair. Therefore, GLP‐1‐based therapies and peroxisome proliferator–activated receptor (PPAR) agonists may restore metabolic communication between adipose tissue and liver and improve regenerative capacity [118, 119].

## 6 Targeted Interventions and Translational Therapies for Liver Regeneration

Self‐regeneration after liver injury represents a fundamental process maintaining hepatic homeostasis and functional recovery. However, clinical translation remains limited by impaired regenerative signaling, metabolic dysregulation, insufficient hepatic targeting, and the limited efficacy of single therapeutic modalities [19].

Emerging regenerative strategies have therefore focused on restoring endogenous repair programs through complementary approaches, including signaling pathway modulation, metabolic reprogramming, targeted delivery systems, immune microenvironment remodeling, gut–liver–brain axis regulation, and cell‐based or tissue engineering therapies. The major therapeutic strategies and their translational framework are summarized in Figure 5.

**FIGURE 5:**
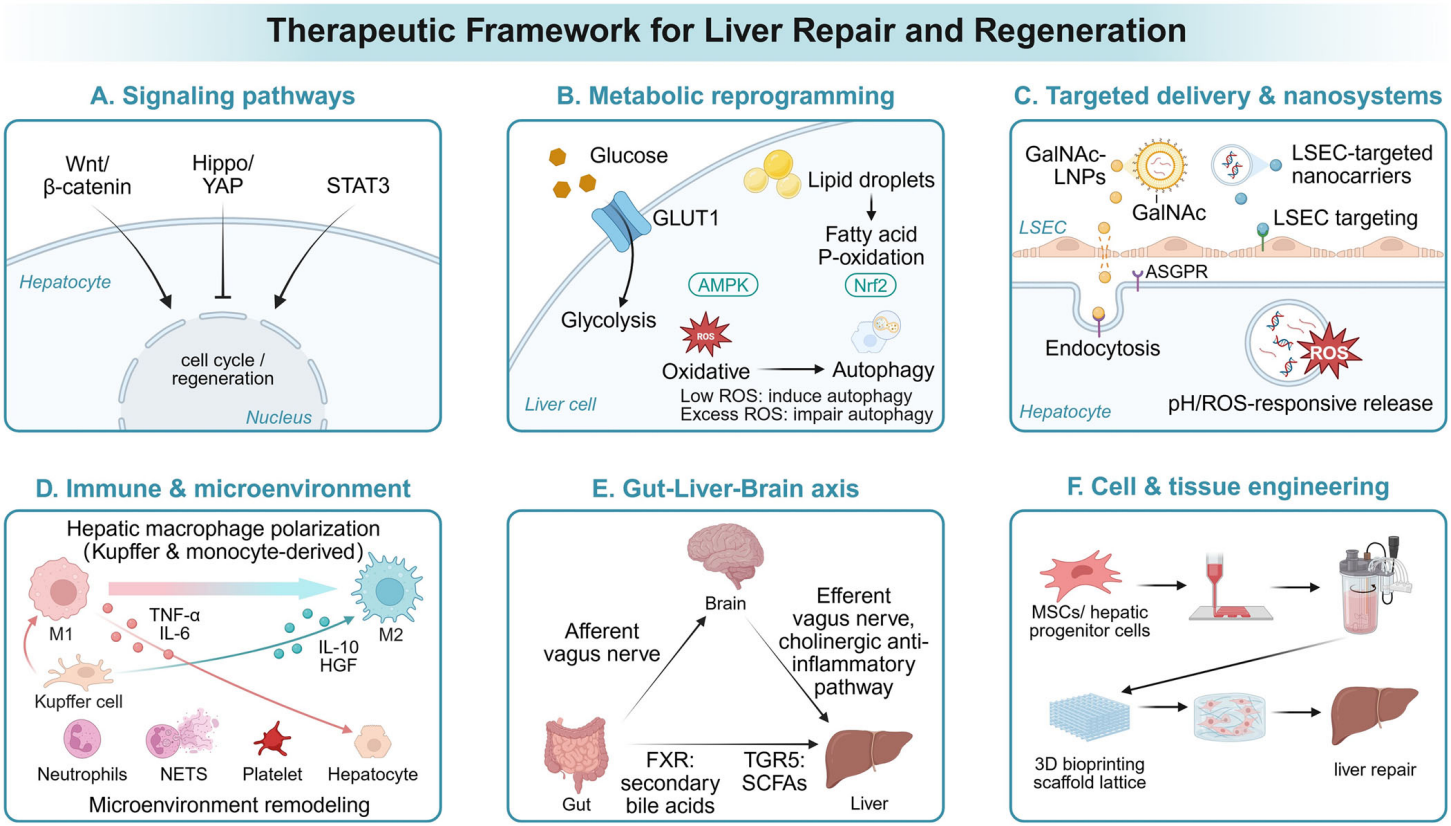
Emerging therapeutic framework for enhancing liver regeneration. Current regenerative strategies target multiple aspects of hepatic repair, including regenerative signaling pathways, metabolic reprogramming, targeted delivery systems, immune microenvironment remodeling, gut–liver–brain communication, and cell‐based tissue engineering. These approaches aim to restore hepatocyte proliferation, metabolic homeostasis, immune balance, and tissue reconstruction through complementary mechanisms, providing potential translational strategies for liver regenerative medicine.

### 6.1 Precise Targeting of Regenerative Signaling Pathways

Liver regeneration depends on coordinated activation and termination of multiple signaling pathways, including Hippo/YAP, STAT3, and Wnt/β‐catenin signaling. Targeting these pathways using small molecules has emerged as a potential strategy to enhance regenerative responses [19].

The Hippo/YAP pathway regulates hepatocyte proliferation and liver size control. During early regeneration, Hippo suppression promotes YAP/TAZ nuclear translocation and activation of proliferation‐associated genes [120]. Current approaches mainly enhance YAP activity through inhibition of upstream kinases such as MST1/2 and LATS1/2. Compounds including XMU‐MP‐1, TRULI, and CAR agonists have shown regenerative effects in experimental models [121, 122]. However, uncontrolled YAP activation may increase tumorigenic risk, highlighting the need for liver‐specific targeting and precise temporal regulation [120].

Other pathways, including STAT3 and Wnt/β‐catenin signaling, exhibit stage‐dependent effects. Moderate activation promotes regeneration after acute injury, whereas persistent activation under chronic inflammatory conditions may contribute to fibrosis progression [123, 124, 125]. Notch and Hedgehog pathways further regulate progenitor cell activation and differentiation, forming an integrated regenerative signaling network [126, 127, 128].

Beyond pathway activation, inhibition of negative regulators represents another therapeutic strategy. MKK4 inhibition by HRX215 reduces the risk of liver failure after extensive hepatectomy, with favorable safety profiles reported in Phase I clinical studies [129]. In addition, chemical small molecule–mediated cellular reprogramming provides a virus‐free strategy to generate hepatocyte‐like cells, offering potential applications for cell replacement therapy [130, 131].

### 6.2 Regulation of Metabolic Reprogramming

Metabolic reprogramming provides essential energy and biosynthetic substrates during liver regeneration. Dynamic regulation of glucose, lipid, and redox metabolism therefore represents an important therapeutic target [2, 132, 133].

During proliferation, hepatocytes shift toward aerobic glycolysis to support rapid ATP production and nucleotide synthesis. GLUT1 and PFK1 participate in this metabolic adaptation and interact with YAP‐dependent proliferative signaling [134, 135]. However, excessive or premature metabolic manipulation may impair regeneration, indicating the importance of stage‐specific intervention [135, 136].

Transient lipid accumulation represents an adaptive response during regeneration, providing substrates for β‐oxidation and membrane synthesis [2]. PPARα and PPARγ exert distinct functions in regulating lipid metabolism and immune responses, whereas abnormal PPARγ activation in hepatocytes may impair regenerative capacity [2, 137, 138, 139].

AMPK and Nrf2 coordinate energy sensing and oxidative stress responses through interaction with mTORC1 signaling, forming an integrated metabolic regulatory network [138, 140]. A dual‐target strategy combining metformin and curcumin has shown potential in improving metabolic dysfunction and promoting regeneration [141, 142]. Future strategies should focus on hepatocyte‐targeted delivery to achieve precise metabolic regulation.

### 6.3 Cell‐Based and Tissue Engineering Approaches for Liver Repair

For severe liver injury and end‐stage liver diseases, pharmacological regulation alone is often insufficient. Cell transplantation, organoid technology, lineage reprogramming, and biomaterial‐based tissue engineering have therefore emerged as alternative regenerative approaches [143, 144].

Mesenchymal stem cells (MSCs) represent one of the most studied cell sources due to their immunomodulatory and paracrine properties. MSCs promote regeneration through mechanisms involving β‐catenin activation and metabolic regulation; however, immune compatibility and long‐term safety remain major challenges [145, 146].

Hepatic progenitor cells and induced pluripotent stem cell (iPSC)–derived hepatocyte‐like cells exhibit regenerative potential in models of acute liver failure and genetic liver diseases [147]. Recent clinical studies using human hepatic progenitor‐like cells (HepLPCs) demonstrated improved liver function and portal hypertension after hepatic artery infusion without immunosuppression, mainly through paracrine mechanisms [148].

Bioartificial liver systems provide temporary support for detoxification and metabolic functions, while advances in 3D bioprinting, gene editing, and ectopic liver regeneration are promoting the transition from cell replacement toward functional tissue reconstruction [149, 150]. Major challenges include vascularization, biliary reconstruction, manufacturing standardization, and long‐term safety evaluation [151, 152].

### 6.4 Targeted Delivery and Nanotechnology‐Based Regulatory Systems

Conventional drugs and nucleic acid therapeutics often exhibit limited hepatic targeting and systemic toxicity. Nanotechnology‐based delivery platforms provide opportunities for controlled accumulation and release within regenerative microenvironments [153, 154].

The unique characteristics of liver sinusoidal endothelial cells and Kupffer cells determine nanoparticle distribution. Particle size, surface charge, and targeting ligands are critical factors affecting delivery efficiency [155]. GalNAc modification targeting ASGPR represents the most established hepatocyte‐targeting strategy, whereas Ly6C‐ and hyaluronic acid–based modifications enable selective delivery to inflammatory macrophages and activated HSCs [156, 157, 158, 159].

Smart nanocarriers responsive to pH, ROS, and enzymatic signals further enable site‐specific release in inflammatory or fibrotic regions [160, 161, 162]. Lipid nanoparticles (LNPs) and engineered exosomes provide efficient delivery of mRNA, siRNA, and functional proteins, showing potential for regenerative pathway modulation [158, 163, 164]. Future development will focus on multi‐target delivery, stage‐specific responsiveness, and improved in vivo stability.

Beyond formulation design, liver regenerative nanomedicines face important regulatory and translational challenges. Regulatory guidance from the US Food and Drug Administration (FDA) and the European Medicines Agency (EMA) emphasize the need for systematic characterization and safety evaluation of nanomaterial‐containing medicinal products, including assessment of biodistribution, accumulation, clearance, and potential immunogenicity [165, 166]. For repeated‐dose administration, these considerations are particularly important because the persistence and clearance of nanomaterials may influence their safety profile and translational development. Incorporating such safety assessments at an early stage of nanocarrier design may help identify potential risks and facilitate the development of liver‐targeted regenerative nanomedicines.

### 6.5 Immunomodulation and Remodeling of the Inflammatory Microenvironment

A balanced inflammatory response is essential for liver regeneration. Early inflammation initiates repair programs, whereas timely resolution prevents fibrosis and regenerative failure [167, 168].

Macrophages are central regulators of this process. Kupffer cells initiate regeneration through inflammatory signaling, followed by macrophage transition toward reparative phenotypes that promote tissue remodeling. Strategies targeting macrophage polarization, including IL‐4, PPARγ agonists, and NLRP3 inhibitors, have shown therapeutic potential [168, 169].

Other immune populations, including neutrophils, γδ T cells, regulatory T cells, innate lymphoid cells, and platelets, contribute to regenerative regulation [167]. IL‐22 represents a multifunctional cytokine with anti‐inflammatory and regenerative properties, whereas cytokine‐targeted therapies require careful consideration of disease stage and context [170].

### 6.6 Regulation of Gut Microbiota and Neuroendocrine Networks

The liver–gut and liver–brain axes integrate microbial metabolites, bile acid signaling, autonomic regulation, and immune responses during liver regeneration. Dysbiosis and neuroendocrine imbalance impair repair through endotoxin accumulation, inflammation, and metabolic disturbance [171, 172].

Probiotics, prebiotics, and fecal microbiota transplantation may restore microbial homeostasis and reduce gut‐derived inflammatory signals [173, 174]. These effects are largely mediated by microbial metabolites, including short‐chain fatty acids and secondary bile acids, which regulate regeneration through metabolic support and FXR/TGR5‐mediated signaling [79, 171].

The autonomic nervous system also regulates regenerative responses [175]. Vagal activation suppresses excessive macrophage activation and promotes repair, whereas sympathetic overactivation may impair hepatocyte proliferation [176, 177].

### 6.7 Multimodal Combination Therapeutic Strategies

Because liver regeneration involves coordinated regulation of signaling, metabolism, immunity, and tissue reconstruction, single‐target therapies are unlikely to achieve comprehensive repair [19, 167].

Current combination strategies integrate pathway modulation, metabolic regulation, cell therapy, nanodelivery, and neuromodulation to enhance efficacy while reducing systemic toxicity [178, 179].

Stage‐specific therapeutic design may further improve precision. Early interventions should enhance regenerative activation, proliferative‐phase strategies should support metabolic demand and tissue expansion, whereas termination‐phase approaches should restore homeostasis and prevent fibrosis [19].

Despite promising advances, challenges remain regarding treatment timing, drug interactions, and clinical trial design [180]. Integration of multi‐omics profiling and artificial intelligence (AI)–based prediction may facilitate individualized regenerative therapies and advance liver regenerative medicine toward precision and intelligent treatment paradigms [179].

We further compare small‐molecule, cell, tissue‐engineered, and nanotherapeutic approaches: Nanocarriers enable precise pathway targeting but carry RES accumulation and immunogenicity risks; small molecules are easy to administer yet lack liver specificity; cell therapy shows strong immunoregulation while suffering low hepatic homing. Multiple preclinical reports hold inconsistent therapeutic effects of hepatic nanomedicines, largely caused by differences in particle design and disease models.

## 7 Clinical Translation of Liver Regeneration: Advances, Challenges, and Future Perspectives

### 7.1 Clinical Progress of Liver Regenerative Therapeutic Strategies

Current clinical development of liver regenerative therapies has evolved into a diversified landscape involving surgical regeneration, small‐molecule therapeutics, cell‐based approaches, tissue engineering, and nanotechnology‐enabled delivery systems. Among these strategies, surgery‐induced regeneration remains the most clinically validated approach, whereas MSC‐based therapies represent the most extensively investigated cell‐based strategy. Meanwhile, small‐molecule agents, bioartificial liver systems, and nanotechnology platforms are gradually transitioning from experimental studies toward clinical translation [129, 181, 182]. Representative ongoing and completed clinical trials in liver injury repair and regeneration are summarized in Table 2.

**TABLE 2:** Representative clinical translation of liver regeneration and liver‐related therapeutics.

Strategy	Intervention type	Target mechanism of action	Indications	Trial phase	Key outcomes	Trial registry ID (NCT number)
Small molecule/biologic	G‐CSF	Mobilizes CD34^+^ stem cells, enhances hepatocyte repair	HBV‐ACLF	Phase III	Improves short‐term survival, but efficacy varies across populations	NCT01502860
Small molecule	MKK4 inhibitor	Inhibits MKK4–JNK pathway, relieves suppression of hepatocyte proliferation	Post‐hepatectomy (CRLM)	Phase I/II	Safety and regenerative potential being evaluated	NCT06213077
Cell therapy	ucMSC	Immunomodulation, anti‐inflammation, paracrine effects	ACLF	Phase III	May reduce mortality and improve liver function (under verification)	NCT04269819
Cell therapy	Autologous BM‐MSC	Paracrine secretion, immunoregulation	Decompensated cirrhosis	Phase II	May improve albumin, bilirubin, MELD (large‐cohort validation needed)	NCT05224960
Cell therapy	Allogeneic AD‐MSC	Paracrine pro‐regenerative factors, anti‐fibrotic	Alcoholic cirrhosis	Phase II	Safe; may improve QoL and partial liver function	NCT02946776
Bioartificial liver	HepaCure (hiHep‐BAL)	Extracorporeal detoxification, metabolism, synthesis	ACLF	Phase I/II	Safety and support function under evaluation	NCT05989958
Surgery	ALPPS	Redirects portal flow, induces rapid remnant hypertrophy	CRLM/complex liver tumors	Clinical	Markedly increases FLR volume, high perioperative risk	—
Surgery	PVE	Embolizes tumor‐bearing lobe, induces compensatory hypertrophy	Extensive hepatectomy	Clinical	Safer than ALPPS, slower hypertrophy	—
Nanodelivery	MTS105 (mRNA‐LNP)	LNP delivers mRNA encoding GPC3 × CD3 BiTE, locally activates T cells	Advanced HCC	Phase I	First human data; dose‐escalation ongoing; preclinical complete tumor regression	NCT06689540

*Note*: NCT numbers are provided for trials registered at https://clinicaltrials.gov. “—” denotes no available trial‐registration identifier for established surgical procedures (ALPPS, PVE) and other interventions.

Despite these advances, clinical trials specifically designed to evaluate liver regeneration remain limited. Most studies primarily assess indirect outcomes, including liver function improvement, MELD score changes, and survival benefits, whereas quantitative evaluation of hepatocyte proliferation and restoration of liver mass remains insufficient [183]. In addition, variations in treatment protocols, patient selection, and outcome assessment criteria have generated substantial heterogeneity among clinical studies, limiting evidence integration. Currently, most regenerative therapies remain in early‐stage clinical development, and large‐scale Phase III trials with robust evidence are still lacking [184, 185].

#### 7.1.1 Development and Clinical Translation of Pro‐Regenerative Small‐Molecule Drugs

Liver regeneration is regulated by multiple signaling pathways, including Wnt/β‐catenin and Hippo/YAP signaling. Small‐molecule regenerative therapies mainly aim to activate pro‐regenerative pathways or inhibit negative regulators of regeneration [129]. However, despite extensive preclinical progress, only a limited number of candidates have entered clinical development.

Among current candidates, HRX215 represents the most advanced regenerative agent [129]. Based on the identification of MKK4 as a negative regulator of liver regeneration, HRX215 demonstrated protective effects against postoperative liver failure in large‐animal models. A Phase I clinical trial confirmed favorable safety and pharmacokinetic profiles, and subsequent Phase Ib/IIa studies are evaluating its efficacy in preventing post‐hepatectomy liver failure and small‐for‐size syndrome. Nevertheless, its effectiveness in patients with chronic liver disease and long‐term safety require further validation.

Other clinically approved metabolic drugs, although not initially designed as regenerative agents, provide alternative translational approaches [186, 187]. Resmetirom, a liver‐targeted thyroid hormone receptor β agonist, improves steatosis, MASH activity, and fibrosis, suggesting that correction of the pathological microenvironment may indirectly restore endogenous regenerative capacity [186].

By contrast, direct modulation of pathways such as Hippo/YAP remains largely restricted to preclinical studies because of potential oncogenic risks associated with uncontrolled activation. Achieving a balance between regenerative enhancement and long‐term safety remains a major challenge for future drug development [181, 188].

#### 7.1.2 Clinical Application of Cell Therapy and Tissue Engineering

Cell‐based therapy represents one of the most extensively investigated approaches in clinical liver regeneration. Increasing evidence indicates that the therapeutic effects of stem cells are mainly mediated through paracrine regulation rather than direct replacement of damaged hepatocytes, shaping current clinical strategies [183, 184].

Among different cell sources, MSC‐based therapies have accumulated the largest clinical experience. Umbilical cord–derived and autologous MSCs have shown potential benefits in patients with ACLF and decompensated cirrhosis; however, the therapeutic effects often decline during long‐term follow‐up, suggesting the need for repeated administration or combination strategies [184].

MSC‐derived extracellular vesicles/exosomes have emerged as promising cell‐free alternatives due to their low immunogenicity and regenerative signaling capacity. However, their clinical application remains at an early exploratory stage [189].

Liver‐derived progenitor cells provide another potential regenerative approach. A prospective open‐label clinical study demonstrated the feasibility and safety of hepatic arterial infusion of HepLPCs in cirrhotic patients, with preliminary improvements in liver function and portal hypertension–related parameters. Nevertheless, larger randomized controlled studies are required to confirm efficacy [148].

Bioartificial liver systems primarily function as bridging therapies for acute liver failure. Although several devices have entered early clinical evaluation, challenges remain regarding coagulation management and functional cell stability [190, 191]. Emerging approaches, including ectopic liver regeneration and 3D bioprinting, represent future directions but remain in early developmental stages [181, 192].

#### 7.1.3 Clinical Exploration of Nanodelivery Systems

Nanodelivery platforms provide opportunities for improving hepatic targeting and controlled release of regenerative therapeutics. However, despite considerable preclinical advances, clinical translation of regenerative nanomedicine remains limited [182].

LNPs represent the most clinically mature platform for nucleic acid delivery. Delivery of regenerative factors such as IL‐22 and HGF mRNA through LNP systems has demonstrated potential for enhancing local therapeutic protein expression. However, challenges related to delivery efficiency, nucleic acid stability, and large‐scale manufacturing remain major barriers [193].

Ligand‐modified nanoparticles targeting HSCs or inflammatory macrophages have shown encouraging preclinical results, but their clinical application requires further validation of targeting specificity and long‐term biosafety [182].

Extracellular vesicle‐based delivery systems combine biological compatibility with endogenous signaling capacity and have entered early clinical exploration. Nevertheless, direct evidence supporting their regenerative efficacy remains insufficient [194, 195].

Overall, successful clinical translation of nanodelivery systems will depend on improving targeting precision, controlled release, manufacturing standardization, and safety evaluation.

#### 7.1.4 Clinical Application and Mechanisms of Surgery‐Induced Liver Regeneration

Compared with pharmacological, cellular, and nanotechnology‐based approaches, surgery‐induced liver regeneration represents the most clinically established regenerative strategy. Its primary application is preoperative enhancement of future liver remnant (FLR) hypertrophy to reduce postoperative liver failure risk [196].

Portal vein embolization (PVE) remains the standard approach for inducing compensatory regeneration before major hepatectomy. By redirecting portal blood flow, PVE stimulates hypertrophy of the FLR and improves functional reserve within several weeks. Mechanistically, PVE‐induced regeneration involves mechanical sensing pathways, including Piezo1 activation and Hippo/YAP signaling [196].

Associating liver partition and portal vein ligation for staged hepatectomy (ALPPS) further accelerates regenerative responses and expands surgical indications for patients with insufficient FLR. Neutrophil‐mediated extracellular matrix remodeling and growth factor release contribute to its rapid regenerative effect. However, increased surgical trauma and complication risks limit widespread adoption [183].

Combined portal vein and hepatic vein embolization provides an alternative strategy for patients with inadequate regeneration after PVE and may enhance regenerative responses without substantially increasing complications [175]. With advances in single‐cell sequencing and spatial profiling, regional differences in regenerative potential are increasingly recognized, providing opportunities for individualized surgical planning in the future.

### 7.2 Major Bottlenecks in Clinical Translation

#### 7.2.1 Pathological Heterogeneity and Limitations of Personalized Therapy

The substantial heterogeneity of liver diseases, reflected by differences in etiology, disease stage, and regenerative capacity, represents a major barrier to clinical translation of regenerative therapies. Current clinical trials generally apply standardized treatment protocols without fully considering disease‐specific regenerative states, limiting the development of precision‐based interventions [197, 198].

For example, the therapeutic effects of MSC‐based immunomodulation observed in alcohol‐associated acute‐on‐chronic liver failure may not be directly applicable to MASH or autoimmune hepatitis, in which metabolic and immune dysregulation represent distinct pathological drivers [197, 199]. Similarly, patients with acute liver injury often retain endogenous regenerative potential, allowing exogenous therapies to enhance intrinsic repair responses, whereas advanced cirrhosis is characterized by extensive loss of parenchymal and vascular structures, which restricts regenerative signaling responses [200].

Therefore, future clinical studies should incorporate disease‐specific stratification strategies and establish regenerative biomarkers to identify patients most likely to benefit from targeted interventions.

#### 7.2.2 Limitations in Targeting Efficiency, Safety, and Long‐Term Efficacy

Insufficient targeting efficiency remains a common limitation among current regenerative therapies. Systemically administered small molecules may cause off‐target effects, whereas transplanted stem cells often exhibit limited hepatic homing due to pulmonary and splenic sequestration. Although intra‐arterial delivery improves hepatic accumulation, its invasive nature limits broad clinical application [197].

Safety concerns represent another major obstacle. Excessive activation of regenerative pathways, such as Wnt/β‐catenin signaling, may increase oncogenic risk, whereas inappropriate modulation of inhibitory pathways may disturb tissue homeostasis. Similarly, iPSC‐derived products require careful monitoring for tumorigenicity, while allogeneic cell therapies remain challenged by immune rejection and long‐term safety concerns [197, 201].

Moreover, the durability of regenerative benefits remains insufficient. Functional improvements after stem cell therapy frequently decline during long‐term follow‐up, whereas bioartificial liver systems mainly provide temporary support rather than permanent restoration [200]. These limitations indicate that future therapies should aim not only to enhance regeneration but also to reconstruct endogenous regulatory networks responsible for sustained tissue homeostasis.

Published data on nanodelivery efficacy remain contradictory across research groups, attributed to variable nanoparticle parameters and experimental conditions, which creates extra translational barriers distinct from small‐molecule and cell therapies.

#### 7.2.3 Lack of Industrialization and Regulatory Evaluation Frameworks

The lack of standardized manufacturing and evaluation systems represents a critical barrier for clinical translation, particularly for cell‐based regenerative therapies. The biological activity of MSC products is influenced by donor characteristics, culture conditions, and manufacturing processes, resulting in substantial batch‐to‐batch variability and challenges in quality control [197].

Furthermore, balancing scalability, accessibility, and safety remains difficult. Autologous cell therapies require individualized preparation and high production costs, whereas allogeneic products provide better scalability but raise concerns regarding immunogenicity and pathogen transmission [202].

Another unresolved issue is the absence of unified regulatory criteria for regenerative therapies. Standardized endpoints, surrogate biomarkers, and long‐term follow‐up requirements specifically designed for evaluating “liver regeneration” are still lacking, limiting clinical comparison and translational development.

### 7.3 Future Directions and Emerging Trends

#### 7.3.1 Multi‐Omics Integration for Identification of Key Regenerative Targets

The integration of AI with multi‐omics technologies is reshaping mechanistic discovery and clinical translation in liver regeneration by facilitating target identification, patient stratification, and therapeutic response prediction [203, 204].

In drug development, deep learning–based models can integrate genomic, transcriptomic, and clinical datasets to identify regenerative targets, optimize candidate compounds, and accelerate virtual screening. For example, AI‐assisted optimization of MKK4 inhibitors may facilitate the development of next‐generation HRX215 derivatives and improve the efficiency of regenerative drug discovery [203].

Furthermore, multi‐omics‐based predictive models may enable individualized assessment of regenerative capacity and therapeutic responsiveness. In cell‐based therapies, AI‐guided identification of potential responders could improve patient selection and enhance clinical trial efficiency [197].

#### 7.3.2 Deciphering the Spatiotemporal Regulatory Networks of Liver Regeneration

Complementary to multi‐omics integration, single‐cell sequencing and spatial transcriptomics have provided unprecedented resolution for defining cellular heterogeneity and spatial organization during liver regeneration. These approaches allow identification of rare regenerative populations and reveal zone‐specific molecular programs within hepatic lobules [205, 206].

Using these technologies, ANXA2^+^ migratory hepatocytes have been identified as a distinct regenerative population characterized by sequential migration toward damaged regions followed by proliferative activation, providing new insights into the spatial dynamics of liver repair [207].

Meanwhile, advanced lineage‐tracing approaches, including ProTracer, enable real‐time analysis of hepatocyte clonal expansion. Recent studies have highlighted the importance of Zone 2 hepatocytes in maintaining hepatic homeostasis and contributing to injury repair, challenging traditional concepts regarding regenerative cell sources [207, 208].

Future integration of genomic, epigenomic, transcriptomic, and metabolomic datasets will facilitate construction of dynamic regenerative networks and enable more precise identification of therapeutic targets and biomarkers for intervention monitoring.

#### 7.3.3 Nanodelivery Platforms Facilitating Translational Advancement of Regenerative Therapies

The future clinical translation of liver regenerative therapies requires integration of biological discoveries with engineering technologies. Emerging platforms, including organ‐on‐chip systems, responsive biomaterials, in situ cellular reprogramming, and standardized manufacturing approaches, provide important tools to overcome current translational barriers.

Liver organ‐on‐chip and microphysiological systems reconstruct three‐dimensional hepatic structures and vascular microenvironments through microfluidic technologies, providing more physiologically relevant platforms for drug screening and mechanistic investigation than conventional models. Integration with patient‐derived iPSCs further enables personalized evaluation of therapeutic responses [209, 210].

In parallel, intelligent biomaterials capable of sensing injury‐associated signals can achieve controlled release of regenerative factors, improving therapeutic precision while reducing systemic exposure [211]. In situ cellular reprogramming and CRISPR‐based genome editing further expand regenerative strategies by enabling conversion of non‐hepatic cells into hepatocyte‐like cells or correction of disease‐associated genetic abnormalities [212].

However, successful clinical implementation requires standardized manufacturing and regulatory frameworks. Development of automated GMP‐compatible production systems, scalable bioartificial liver platforms, and reproducible small‐molecule manufacturing processes will be essential for translating regenerative technologies into clinical practice [213].

## 8 Conclusions and Future Perspectives

Liver regeneration represents a unique biological capability and provides a fundamental basis for developing regenerative therapies for liver diseases. This review systematically integrates multi‐scale research progress ranging from basic molecular mechanisms to clinical translational practice, sorting out core regulatory signaling cascades, multicellular niche crosstalk, metabolic reprogramming, and multi‐organ intercommunication networks. We also comprehensively summarize cutting‐edge therapeutic modalities, including small‐molecule targeted agents, cell and tissue engineering therapy, nanomedicine delivery platforms, and surgery‐triggered compensatory liver regeneration.

Despite abundant preclinical achievements, the clinical transformation of liver regenerative therapy faces prominent barriers, among which regenerative heterogeneity is the core limiting factor. Differences in disease etiology, progression stage, and intrinsic hepatic regenerative capacity drastically affect therapeutic efficacy [197]. Most current clinical trials adopt unified intervention schemes without patient stratification, resulting in inconsistent treatment outcomes. We therefore hold that classification of patients relying on regenerative biomarkers is a necessary premise to stabilize clinical curative effects.

From our perspective, future liver repair therapies should abandon single‐target monotherapy and turn to stage‐adapted multimodal combined regimens. Isolated pathway modulation fails to match the complex regulatory network of liver regeneration. Combined strategies that simultaneously tune signal pathways, cellular metabolism, immune microenvironment, and targeted delivery systems can synergistically boost endogenous repair while lowering systemic off‐target side effects.

Multi‐omics profiling and AI will serve as core enablers for precision liver regeneration. Omics data help identify hidden regenerative biomarkers and key regulatory targets, while AI models predict individual repair potential, screen eligible patients, and optimize trial protocols to overcome empirical treatment limitations. Overall, liver regenerative medicine is shifting from simply enhancing hepatocyte proliferation to addressing three core questions: who requires intervention, when treatment should start, and how to accurately control repair strength. Solving regenerative heterogeneity, improving tissue targeting, and building personalized regimens will drive major breakthroughs. Cross‐disciplinary integration of molecular biology, hepatology, bioengineering, and computational science will speed up the clinical translation of liver regeneration theories, and the regulatory rules summarized herein can also guide regenerative research on other solid organs.

## Author Contributions

M.P. and N.A. conceptualized, investigated, and wrote the article. Y.F. visualized and investigated the article. Y.L.H., C.P., Y.S., Y.Z.H., T.L., Q.W., Q.S., P.L., X.L., and W.Y. investigated and reviewed the article. Z.H., X.L., and J.L. conceptualized, supervised, and reviewed the article. J.L. administered the project. M.P. and N.A. made equal contributions to the manuscript. All authors have read and approved the final manuscript.

## Ethics Statement

The authors have nothing to report.

## Conflicts of Interest

The authors declare no conflicts of interest.

## Data Availability

This review relies on published literature; no original experimental data were generated.
